# Ameliorating Effect of *Crassocephalum rabens* (Asteraceae) Extract on Skin Aging: A Randomized, Parallel, Double-Blind, and Placebo-Controlled Study

**DOI:** 10.3390/nu14132655

**Published:** 2022-06-27

**Authors:** Chen-Meng Kuan, Chia-Hua Liang, Wei-Hsiu Chuang, Ting-Yu Lin, Pang-Kuei Hsu

**Affiliations:** 1 Department of Research and Development, Greenyn Biotechnology Co., Ltd., Taichung City 40768, Taiwan; michellechuang@greenyn.com.tw (W.-H.C.); franklin@greenyn.com.tw (T.-Y.L.); leon@greenyn.com.tw (P.-K.H.); 2 Department of Cosmetic Science, Chia Nan University of Pharmacy and Science, Tainan City 71710, Taiwan; tinna_ling@mail.cnu.edu.tw

**Keywords:** *Crassocephalum rabens*, Asteraceae, anti-aging, dLGG, skin health, dietary supplement

## Abstract

*Crassocephalum rabens* (Asteraceae) is a common herb used in Taiwanese folk medicine to treat inflammation-related syndromes. Pharmacological studies have revealed that galactolipids exhibit anti-oxidative, anti-inflammatory, and anti-hyaluronidase activities and improve skin wrinkles, moisture, and elasticity in healthy subjects. However, the anti-aging effects of *C. rabens* and its primary active compound, 1,2-di-O-linolenoyl-3-O-β-galactopyranosyl-sn-glycerol (dLGG), remain elusive. Here, we investigated whether *C. rabens* can improve skin conditions in healthy individuals using a double-blind approach. Forty enrolled volunteers were randomly and equally assigned to the control or treatment group and were required to take either a placebo or a *C. rabens* extract capsule daily for one month. Skin parameters were measured before and after the study. The results showed significant differences in skin elasticity, wrinkles, collagen content, brightness, and hydration between the baseline and week 4 in the treatment group. Particularly, compared with those in the placebo group, skin wrinkles (*p* < 0.05), brightness (*p* < 0.001), collagen content (*p* < 0.01), and UV spots (*p* < 0.05) were notably improved after treatment with the *C. rabens* extract. Our study successfully demonstrated the application of *C. rabens* in preventing skin aging. Further investigations will be conducted to study the underlying anti-aging mechanism of dLGG.

## 1. Introduction

The skin is the largest organ in human body, accounting for approximately 15% of the total adult body weight [1]. The skin protects the human body from direct environmental impact and aids in thermoregulation and osmoregulation [2]. Skin health may represent the overall well-being of humans; therefore, the use of cosmetic or dietary supplements with functional ingredients has become a popular way to slow skin aging and maintain good skin conditions [3,4,5]. Skin aging is associated with increased oxidative stress in skin cells, elicited by internal (e.g., genes and cellular metabolism) and external (e.g., air pollutants, smoking, and ultraviolet (UV) radiation) factors [6]. In particular, 80% of premature aging (photoaging) results from chronic exposure to UV radiation (i.e., UVA and UVB) [7,8]. Aged skin is generally characterized by laxity, roughness, wrinkles, dullness, pigmentation, and a thickened epidermis [9]. Exposure to UV radiation leads to the production of reactive oxygen species (ROS) and increases the expression of transcription factor activator protein 1 (AP-1) via activation of the mitogen-activated protein kinase (MAPK) signaling pathway [10]. The upregulation of AP-1 increases the expression of matrix metalloproteinases (MMPs) and inhibits collagen expression in fibroblasts; the degradation of collagen leads to the formation of wrinkles [11]. Collagen is present in 70% of the dermis and aids in the stabilization of the extracellular matrix structure [12]. In addition, ROS cause DNA, lipid, and protein oxidation while driving melanogenesis in melanocytes [13].

*Crassocephalum rabens* (Asteraceae), also known as Zhaohe Cao, is a common herbal plant that is conventionally used as a folk remedy to treat inflammation-related syndromes in Taiwan [14]. Studies have revealed that *C. rabens* possesses anti-inflammatory and anti-cancer activities [14,15,16,17,18]. The prominent active compound of *C. rabens* is 1,2-di-O-linolenoyl-3-O-β-galactopyranosyl-sn-glycerol (dLGG), a phytogalactolipid that exhibits anti-oxidative, anti-tumor, anti-inflammatory, and hepatoprotective effects in cells and rodents [15,16,17,18,19,20]. A study showed that dLGG effectively attenuates the recurrence of triple-negative breast cancer and lung metastasis through the downregulation of fatty acid-binding proteins, peroxisome proliferator activated receptor γ, and epoxyeicosatrienoic acids [21]. Furthermore, Takahashi *et al.* demonstrated that dLGG scavenges free radicals in promyeloblasts [20]. Moreover, botanical extracts (e.g., *Rosa canina*) containing galactolipids (e.g., monogalactosyl diacylglycerol and digalactosyl monoacylglycerol) have been shown to delay skin aging and ameliorate skin conditions in *in vitro* and clinical studies [22,23,24,25,26,27,28].

However, to the best of our knowledge, the anti-aging effects of *C. rabens* have not been evaluated in detail. The present study investigated the effects of *C. rabens* extract on healthy skin using a randomized, parallel, double-blind, and placebo-controlled approach.

## 2. Materials and Methods

### 2.1. Preparation of C. rabens Extract

The voucher specimen for *C. rabens* refers to the specimen no. 21152, 33768, 36303, 38867, 38959 in the Taiwan Wild Plant Database [29]. The aerial parts of *C. rabens* (harvested approximately 120 days after seeds were sown) were cleaned with running water and distilled water. The samples were dried using a food dehydrator at 40 °C, followed by crushing with a pulverizer. The powder was processed via ultrasonic extraction with 95% ethanol (1:10 w/v) at 40 °C for 3 h, and the extract was filtered using Whatman filter paper No. 1. The filtrate was freeze-dried and stored in a freezer. For this study, the *C. rabens* powder was filled in capsules.

### 2.2. dLGG Analysis

The levels of dLGG were analyzed using a high-performance liquid chromatography (HPLC) system (e2695; Waters Corporation, Milford, MA, USA) equipped with a photodiode array detector (2998; Waters Corporation) and an Inertsil ODS-HL analytical column (4.6 × 250 mm, 5 μm; GL Sciences Inc., Tokyo, Japan). The flow rate of the mobile phase (98% of methanol) was 1.0 mL/min, and the column temperature was set at 30 °C. The injection volume was 10 μL, and the detection wavelength was 210 nm.

### 2.3. Study Design

This clinical study was approved by the Ethics Committee of the Antai Medical Care Corporation, Antai Tian-Sheng Memorial Hospital (IRB No. 21-129-A), and the study protocol was registered with ClinicalTrials.gov (NCT05309161). This study was performed in accordance with the principles of the Declaration of Helsinki, and all subjects provided written informed consent before the study. Forty healthy subjects were enrolled in this study, and all subjects completed the study. Eligible participants were healthy adults over 20 years of age. The exclusion criteria were as follows: (i) non-volunteers; (ii) skin disorders; (iii) liver or kidney diseases; (iv) allergy to cosmetics, drugs, or food supplements; (v) pregnancy and lactation; (vi) acceptance of esthetic medicine treatments (e.g., intense pulse light, medical peelings, or laser therapy) before 4 weeks of the study; (vii) suffering from chronic or severe diseases; (viii) members of this research group.

The study had a randomized (1:1 ratio), double-blind, parallel, and placebo-controlled design. The subjects were instructed to take a maltodextrin capsule (placebo; 180 mg) or a *C. rabens* extract capsule (treatment; 180 mg) every day for 4 weeks. Skin parameters were recorded at weeks 0 and 4.

### 2.4. Efficacy Analysis

The number of skin pores, spots, UV spots, and brown spots and wrinkles, red areas, and the presence of texture on the full face (i.e., forehead, orbital rim, upper and lower cheeks, chin, and nose) were measured using the VISIA^®^-CR skin analysis system (Canfield, Parsippany-Troy Hills, NJ, USA). Skin lightness of the upper cheek was measured using Chroma Meter MM-500 (Minolta, Osaka, Japan). Skin hydration content of the upper cheek was measured using Corneometer^®^ CM825 (Courage + Khazaka Electroni, Cologne, Germany), and the final value was the average of triplicate measurements. The skin elasticity of the upper cheek was measured using Soft Plus (Callegari 1930, Parma, Italy), and the final value was the average of triplicate measurements.

### 2.5. Statistical Analysis

Data were presented as mean values and standard deviations. The age comparison and measured results of skin parameters within groups and between groups were analyzed using a paired *t*-test and an independent *t*-test, respectively, with Excel 2021 (Microsoft, Redmond, WA, USA), and *p* < 0.05 was considered statistically significant.

## 3. Results

### 3.1. dLGG Analysis

**Figure 1:**
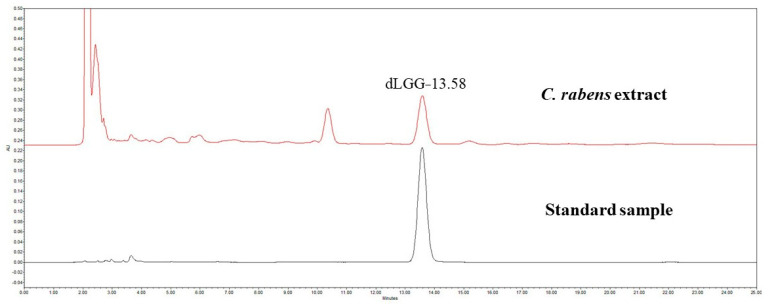
HPLC analysis for dLGG in the *C. rabens* extract.

*C. rabens* contains a variety of phytochemicals, but dLGG is its most well-known marker compound, as evidenced by several pharmacological studies [12,13,14,15,16,17,18]. In the HPLC analysis, dLGG was detected in the *C. rabens* extract at 13:58 min based on the running time of the standard sample, and its concentration was determined to be 218.05 mg/mL (Figure 1).

### 3.2. Baseline Characteristics

Forty subjects were recruited for this study and randomly assigned to the placebo or treatment group, with all participants completing the study (Table 1). The male-to-female ratio was identical for both groups, with mean ages of the placebo and treatment groups being 46.4 and 43.9 years, respectively, and no statistical difference in the mean age between the groups.

### 3.3. Improvement in Collagen-Associated Parameters

Table 2 displays the results of collagen-associated parameters before and after the study. No significant differences in skin pores were observed between baseline and week 4 for both the groups. The mean change (Δ) in skin pores in the treatment group was three times greater than that in the control group; however, the difference between the groups was not obvious. The mean changes in skin elasticity were similar for both groups, whereas both the placebo and *C. rabens* extract led to a significant increase in skin elasticity at week 4.

The mean changes in skin texture in the placebo and treatment groups were +19.2 and −12.8, respectively, while there were no significant differences in skin texture within and between groups. In contrast, a noticeable reduction in wrinkles was observed in the treatment group. The mean changes in skin wrinkles in the placebo and treatment groups were +0.1 and −3.8, respectively. The improvement effect was weighted over placebo effects. Moreover, both groups exhibited significantly positive increments in collagen content after the study; nevertheless, the *C. rabens* extract showed a more prominent improvement in collagen synthesis than the placebo.

### 3.4. Improvement in Skin Pigmentation

Table 3 shows the number of measured skin spots, UV spots, and brown spots before and after the study. Skin spots refer to visible skin marks, UV spots represent epidermal melanin that can absorb UV light, whereas brown spots represent pigmentation on and beneath the skin. No significant changes in the number of skin spots or brown spots were observed within or between the groups. The difference in the number of UV spots between weeks 0 and 4 was –12.8 in the treatment group, but the mean value of the number of UV spots at week 4 increased by 7.5 relative to the baseline result. The comparison of UV spots between the groups was significant.

### 3.5. Other Skin Parameters

Table 4 shows the measured results of skin brightness, hydration, and red areas before and after the study. *C. rabens* extract significantly improved the skin brightness of subjects over 4 weeks, and the improvement effect could be distinguished from the placebo effect. In addition, there was a significant improvement in skin hydration between the baseline and week 4 in the treatment group. The improvement in red areas after treatment with the *C. rabens* extract was not obvious.

## 4. Discussion

The safety of the *C. rabens* extract has been verified by acute and sub-acute toxicity studies in rats, and the no-observed-adverse-effect-level (NOAEL) was reported to be greater than 1666.7 mg/kg body weight in male and female rats [30]. Accordingly, the acceptable daily intake (ADI) of the extract for a 60 kg adult is approximately 1 g, based on a 100-fold safety factor [31]. Our intervention dose was 180 mg every day, which is 5.5 times lower than the ADI.

While little is known regarding the mechanisms involved in the improvement of skin health, a few *in vitro* and clinical studies point towards the role of galactolipids in slowing down skin aging [22,23,24,25,26,27,28]. Rose (*R. canina*) hips, which contain abundant galactolipids (e.g., (2S)-1,2-di-O-[(9Z,12Z,15Z)-octadeca-9-12-15-trienoyl]-3-O-β-d-galactopyranosyl glycerol), inhibit the expression of proinflammatory cytokines (e.g., tumor necrosis factor α and interleukins 1β and 6) and proinflammatory enzymes (e.g., MMPs and cyclooxygenase-2) while simultaneously reducing oxidative stress in cells [22,23]. The galactolipids isolated from *Impatiens parviflora* DC exhibit anti-hyaluronidase activity [24], while *M. integrifolia* leaf extract, which is rich in monogalactosyl diacylglycerol 36:4 and digalactosyl monoacylglycerol 18:2, exerts a strong tyrosinase inhibitory activity [25]. Furthermore, clinical studies have shown that *R. canina* hips can significantly improve skin wrinkles, hydration, and elasticity in healthy individuals [26,27]. A wheat polar lipid complex extract containing digalactosyl diglycerides notably increased skin hydration, elasticity, and smoothness while reducing trans-epidermal water loss, skin roughness, and skin wrinkles in healthy subjects after a month of treatment [28].

No adverse effects were observed throughout this study. After the 4 week intervention, the scores of skin elasticity, wrinkles, and collagen content in subjects were significantly improved in comparison with the baseline. The *C. rabens* extract reinforced skin elasticity and reduced skin wrinkles as compared with the placebo. Contrastingly, although the mean changes in skin pores and texture reached a certain degree of reduction, individual variations affected the outcomes. We propose that these results may be attributed to the anti-aging effects of galactolipids. Galactolipids are powerful anti-oxidants and anti-inflammatory agents that can effectively inhibit collagenase and hyaluronidase activities [22,23,24]. An excessive ROS production and the expression of pro-inflammatory cytokines exacerbate the degradation of collagen and elastin in the skin owing to the upregulation of MMPs and downregulation of collagen synthesis in fibroblasts, which undermines the skin structure and promotes the formation of skin wrinkles [32]. In addition, hyaluronic acid (HA), comprising D-glucuronic acid and N-acetyl-D-glucosamine, plays an essential role in the integration and retention of water in skin tissue [33]. ROS cause the degradation of HA and activation of hyaluronidase, consequently leading to dry and rough skin and increased skin wrinkles [34]. The anti-hyaluronidase activity of the *C. rabens* extract might partially be responsible for the significant improvement in skin hydration of the subjects (Table 4).

Regarding depigmentation, the *C. rabens* extract significantly reduced the number of UV spots (melanin in the basal and suprabasal layers of the epidermis) caused by sun damage, but its efficacy in reducing visible skin marks in the dermis was not remarkable [35]. We hypothesized that the positive changes in the appearance of spots and brown spots might be more pronounced if the extract was consumed for longer periods of time. This speculation was based on the anti-tyrosinase activity of galactolipids and the corresponding observation of a small improvement in spot appearance (Table 3) [25].

Skin brightness is related to skin tone, hydration, and surface roughness. The significant change in skin brightness was correlated with the complex effects of improvements in skin spots, texture, pores, and hydration. Skin hydration was remarkably improved in the treatment group, suggesting that dLGG could reduce oxidative stress and suppress hyaluronidase activity in skin cells [24]. Red areas result from sensitive skin, winter xerosis, alcohol ingestion, and UV radiation [36]. Since we did not recruit individuals with sensitive skin and conducted this study in spring, the study conditions might explain the insignificant change in red skin areas in the treatment group.

Our study suggests that *C. rabens* can be developed as a dietary supplement to improve skin health and delay skin aging; however, there are several limitations to this study. First, it is difficult to extrapolate the results to men, considering the disproportional female-to-male ratios in both groups. Second, the addition of a positive control, different concentrations of *C. rabens* extracts, more parameters (e.g., temperature, pH value, erythema, trans-epidermal water loss, stratum corneum hydration, and stiffness), weekly measurement, and follow up were not considered in this study, which might have slightly affected the accurate observation of the anti-aging activity of the *C. rabens* extract. Third, the underlying mechanism of dLGG in skin aging is unclear and has not been meticulously verified in this study; we speculate that the improvement in skin health by the extract is likely due to the synergistic effect of phytochemicals present in *C. rabens*. We will conduct further investigations to examine the anti-aging effects of dLGG in cells and rodents (including skin disease models). Finally, we will consider the shortcomings and further animal results and conduct another clinical study to strictly verify the anti-aging activity of *C. rabens* in terms of dietary supplements and cosmetics (in the form of gel or cream).

## 5. Conclusions

In summary, we successfully demonstrated the application of *C. rabens* in preventing skin aging. However, considering the limitations of the study design, further investigations should be carried out to verify the anti-aging activities of *C. rabens* and dLGG in rodents and humans.

## Figures and Tables

**Table 1:** Baseline characteristics (mean value ± SD).

	Placebo (*n* = 20)	Treatment (*n* = 20)	*p*-Value
Male/female participants	2/18	2/18	N/A
Mean age (years)	46.4 ± 13.22	43.9 ± 11.9	0.533

**Table 2:** Measurement of collagen-related factors in the subjects (mean value ± SD).

Index	Group	Measurement	Week 0	Week 4	*p*-Value
Pores	Placebo	Original	718.4 ± 419.4	708.4 ± 394.8	0.72 ^a^
		Δ		−10.1 ± 127.0	
	Treatment	Original	731.1 ± 371.7	696.5 ± 335.6	0.18 ^a^, 0.92 ^b^
		Δ		−34.6 ± 111.1	0.52 ^b^
Elasticity	Placebo	Original	50.5 ± 3.0	51.3 ± 2.7 ***	<0.001 ^a^
		Δ		0.8 ± 1.1	
	Treatment	Original	50.3 ± 2.8	51.4 ± 3.1 ***	<0.001 ^a^, 0.91 ^b^
		Δ		1.1 ± 1.4	0.52 ^b^
Texture	Placebo	Original	548.4 ± 402.5	567.6 ± 446.8	0.38 ^a^
		Δ		19.2 ± 96.5	
	Treatment	Original	434.2 ± 319.8	421.4 ± 304.9	0.40 ^a^, 0.25 ^b^
		Δ		−12.8 ± 66.4	0.23 ^b^
Wrinkles	Placebo	Original	16.7 ± 13.3	16.8 ± 13.8	0.94 ^a^
		Δ		0.1 ± 6.2	
	Treatment	Original	12.7 ± 9.6	9.0 ± 8.4 ***	0.002 ^a^, 0.04 ^b^
		Δ		−3.8 ± 4.6 ^#^	0.03 ^b^
Collagen content	Placebo	Original	58.3 ± 20.8	60.2 ± 20.1 *	0.04 ^a^
		Δ		1.9 ± 3.9	
	Treatment	Original	53.1 ± 23.4	58.2 ± 22.8 ***	<0.001 ^a^, 0.78 ^b^
		Δ		5.2 ± 1.5 ^###^	0.001 ^b^

^a^ Comparison within groups; ^b^ comparison between groups; *, ^#^ *p* < 0.05; ***, ^###^ *p* < 0.001. The asterisk and hashtag symbols indicate statistical significance within and between groups, respectively. Δ Represents the difference between weeks 0 and 4.

**Table 3:** Measurement of skin pigmentation in the subjects (mean value ± SD).

Index	Group	Measurement	Week 0	Week 4	*p*-Value
Spots	Placebo	Original	120.6 ± 48.4	120.8 ± 51.2	0.96 ^a^
		Δ		0.2 ± 14.9	
	Treatment	Original	101.6 ± 30.9	97.5 ± 28.2	0.21 ^a^, 0.08 ^b^
		Δ		−4.2 ± 14.3	0.36 ^b^
UV spots	Placebo	Original	349.9 ± 56.9	357.4 ± 58.3	0.15 ^a^
		Δ		7.5 ± 22.3	
	Treatment	Original	347.7 ± 51.2	335.5 ± 50.6	0.08 ^a^, 0.21 ^b^
		Δ		−12.2 ± 29.2 ^#^	0.02 ^a^
Brown spots	Placebo	Original	320.5 ± 101.7	320.5 ± 100.5	1.00 ^a^
		Δ		0 ± 17.0	
	Treatment	Original	321.1 ± 78.4	311.9 ± 77.9	0.06 ^a^, 0.76 ^b^
		Δ		−9.2 ± 20.6	0.13 ^b^

^a^ Comparison within groups; ^b^ comparison between groups; ^#^ *p* < 0.05. The hashtag symbol indicates statistical significance between the groups. Δ Represents the difference between weeks 0 and 4.

**Table 4:** Measurement of skin brightness, skin hydration, and red areas in the subjects (mean value ± SD).

Index	Group	Measurement	Week 0	Week 4	*p*-Value
Brightness	Placebo	Original	57.9 ± 3.1	58.0 ± 3.1 ***	<0.001 ^a^
		Δ		0.2 ± 0.1	
	Treatment	Original	58.3 ± 2.6	59.5 ± 2.7 ***	<0.001 ^a^, 0.12 ^b^
		Δ		1.2 ± 1.0 ^###^	<0.001 ^b^
Hydration	Placebo	Original	40.4 ± 8.8	40.7 ± 8.1	0.29 ^a^
		Δ		0.4 ± 1.4	
	Treatment	Original	39.8 ± 8.5	40.9 ± 8.4 ***	0.02 ^a^, 0.95 ^b^
		Δ		1.1 ± 1.8	0.18 ^b^
Red areas	Placebo	Measurement	190.1 ± 78.2	183.1 ± 74.9	189.2 ± 92.2
		Δ		−7.0 ± 25.1	−0.9 ± 27.3
	Treatment	Measurement	157.9 ± 64.6	146.3 ± 59.6	153.5 ± 61.0
		Δ		−11.6 ± 18.1	−4.4 ± 28.3

^a^ Comparison within groups; ^b^ comparison between groups; ***, ^###^ *p* < 0.001. The asterisk and hashtag symbols indicate statistical significance within and between groups, respectively. Δ Represents the difference between weeks 0 and 4.

## Data Availability

Not applicable.
